# *N*,*N'*-Dinitrosopiperazine–Mediated Heat-Shock Protein 70-2 Expression Is Involved in Metastasis of Nasopharyngeal Carcinoma

**DOI:** 10.1371/journal.pone.0062908

**Published:** 2013-05-07

**Authors:** Zhengke Peng, Na Liu, Damao Huang, Chaojun Duan, Yuejin Li, Xiaowei Tang, Wenhua Mei, Feng Zhu, Faqing Tang

**Affiliations:** 1 Medical Research Center and Clinical Laboratory, Zhuhai Hospital, Jinan University, Zhuhai People’s Hospital, Zhuhai, People’s Republic of China; 2 Medical Research Center and Clinical Laboratory, Xiangya Hospital, Central South University, Changsha, People’s Republic of China; 3 Metallurgical Science and Engineering, Central South University, Changsha, People’s Republic of China; 4 Hormel Institute, University of Minnesota, Austin, Minnesota, United States of America; Karolinska Institutet, Sweden

## Abstract

*N*,*N*′-Dinitrosopiperazine (DNP) is invovled in nasopharyngeal carcinoma (NPC) development and metastasis, and it shows organ specificity to the nasopharyngeal epithelium. Herein, we demonstrate that DNP induces heat-shock protein (HSP) 70-2 expression in NPC cells (6-10B) at a non-cytotoxic concentration. DNP induced HSP70-2 expression in a dose- and time- dependent manner, but showed no effect on other HSP70 family members. Furthermore, DNP also increased *HSP70-2* RNA transcription through directly binding to the hypoxia-responsive elements (HRE) and heat shock elements (HSE) located in the *HSP70-2* promoter. DNP-mediated *HSP70-2* expression might act through enhancing the transcription of *HSP70-2* RNA. Importantly, DNP induced motility and invasion of 6-10B cells dose- and time-dependently, and DNP-mediated NPC metastasis was confirmed in nude mice, which showed high HSP70-2 expression in the metastatic tumor tissue. However, the motility and invasion of NPC cells that were stably transfected using short interfering RNA against HSP70-2 could not effectively induce DNP. These results indicate that DNP induces HSP70-2 expression through increasing *HSP70-2* transcription, increases the motility and invasion of cells, and promotes NPC tumor metastasis. Therefore, DNP mediated *HSP70-2* expression may be an important factor of NPC-high metastasis.

## Introduction

Nasopharyngeal carcinoma (NPC) is endemic in certain regions of the world and is particularly high in Southeast Asia, with an incidence of 30–80 cases per 100,000 people per year in southern China [1]. NPC exhibits highly invasive and metastatic features, and approximately 90% of patients show cervical lymph node metastasis at the time of initial diagnosis [2]. Failure of therapies to treat advanced NPC have resulted in high rates of local recurrence as well as distant metastasis [3]–[5]. Moreover, the underlying mechanism behind NPC metastasis remains unclear.

Previous studies have shown that DNP induces rat nasopharyngeal carcinogenesis, which enhances HSP70 expression [6], and rats with high DNP concentrations exhibit high metastasis rates. This implies that HSP70 may be associated with NPC metastasis. Generally, HSP70 family is composed of HSP70-2, HSP70t, HSC70, GRP75, GRP78 (HSP70-5), and HSP70-4. These proteins function in a variety of roles; they act as molecular chaperones facilitating the assembly of multiprotein complexes, participate in the translocation of polypeptides across cell membranes and to the nucleus, and aid in the proper folding of nascent polypeptide chains. Further, HSP70-2 plays an important role in protein-protein interactions that result in proper folding, confirmation, transport, and assembly of proteins in the cytoplasm, mitochondria, and endoplasmic reticulum [7], [8]. HSP70-2 is overexpressed in various cancer cell lines [9] and is involved in the growth, migration, and invasion of cancer cells [10]. Further, HSP70t interacts with pre-protein or mature forms of organelles. HSP90s form chaperone complexes and perform functional roles [11]. HSC70 is an ATP-dependent molecular chaperone which binds unfolded proteins, and participates in various cellular processes such as *de novo* protein folding, protein translocation across organelle membranes, and uncoating of clathrin-coated vesicles [12], [13]. GRP75, glucose-regulated protein, has been localized to various cellular compartments, including mitochondria, endoplasmic reticulum, and cytoplasmic vesicles, and has multiple functions ranging from stress response, intracellular trafficking, antigen processing, control of cell proliferation, differentiation, and tumorigenesis [14]. GRP78, glucose-regulated protein, is also known as HSP70-5 and is required for adipocyte differentiation, glucose homeostasis, and maintaining a balance of secreted adipokines. GRP78 is implicated in the integration of cellular signaling pathways, including the unfolded protein response, apoptosis, and autophagy to determine cell fate in response to antiestrogen therapy [15]. HSP70-4 is expressed during normal lens development in the eye. Embryonic HSP70-4 expression is also activated in a cell-specific manner following heavy metal exposure [16].

HSP70-2 is constitutively expressed at low levels in most tissues, but at high levels in the testis and brain [17]. The *HSP70-2* gene is located on chromosome 14, and its encoded protein shows 84% amino acids sequence homology to HSP70 [7]. HSP70-2 has previously been assigned a particular function in male germ cells, specifically, it is essential for formation of the active CDC2/cyclin B complex during metaphase of the first meiotic division of male germ cells [18], [19]. Many studies have focused on the intracellular role of HSP70-2 in tumorigenesis [20]–[22], and have shown that HSP70-2 is associated with NPC development [23]. Recent data have indicated that HSP70-2 is upregulated in metastatic cancers and its expression promotes cancer metastasis [9]. However, the molecular mechanisms underlying HSP70-2 upregulation and its function in tumor metastasis remain unclear.

In studies of Chinese populations, a region of high-incidence, the relative NPC risk is associated with weekly salt-preserved fish consumption. Typical daily consumption generally ranged from 1.4 to 3.2, whereas daily consumption in the regions of high incidence ranged from 1.8 to 7.5 [24]–[29]. The process of salt preservation is inefficient, some bacteria can induce conversion of nitrates into nitrites, forming carcinogenic *N*-nitroso compounds [30]. *N*,*N*′-Dinitrosopiperazine (DNP) accumulates in the human body when salted fish is consumed [28], [31], [32]. DNP can induce NPC through high expression of HSP70 [6], which results in a high incidence of metastasis. In clinical assays, NPC patients with lymph nodes metastasis exhibit high DNP serum levels compared to patients without metastasis. DNP promotes NPC cell motility and invasion *in vitro* and induces metastasis in nude mice [33]. Here, we demonstrate a novel view of the mechanism behind NPC metastasis, that is, DNP contributes to metastasis of NPC through induction of HSP70-2 expression.

## Materials and Methods

### Ethics Statement

The present study protocol was approved by the ethical committee at Zhuhai Hospital of Jinan University and Xiangya Hospital of Central South University, China.

### Reagents and Antibodies

DNP was donated by the Cancer Research Institute of Central South University. Its boiling point, melting point, and other chemical characteristics were all determined to be within an acceptable range [6]. Chemical reagents, including Tris, HCl, SDS (sodium dodecyl sulfate), Na_2_S_2_O_3,_ K_3_Fe(CN)_6_, TPCK-Trypsin, NH_4_HCO_3_, acrylamide, urea, thiourea, NP-40, Triton X 100, DTT, PMSF, CHAPS, EDTA, and pharmolyte were purchased from Sigma-Aldrich (St. Louis, MO) [34]. Reagents for the lactate dehydrogenase **(**LDH) assay were purchased from Autec Diagnostica Co (Botzing, Germany). Antibodies against HSP70, HSP70-2, HSP70t, HSC70, GRP75, and GRP78 were purchased from Santa Cruz Biotechnology, Inc. (Santa Cruz, CA). HSP70-4 antibody was purchased from Abcam (Cambridge, MA, UK). HSP70 antibody is a mouse monoclonal antibody raised against HSP70 and purified from HeLa cells with epitope mapping to amino acids 436–503 of human HSP70. HSP70-2 antibody was raised against the amino acid sequence SKLYQGGPGGGGSSGGPT (position 611–628) of the human HSP70-2. The HSP70t polyclonal antibody was affinity purified from rabbit and raised against a synthetic HSP70tL peptide of human origin. The HSC 70 antibody is a mouse monoclonal antibody specific for the epitope mapping to amino acids 580–601 at the C-terminus of the human HSC 70 protein. The GRP75 antibody is a rabbit polyclonal antibody raised against human GRP 75 amino acids 525–679. The GRP78 rabbit polyclonal antibody was raised against amino acids 525–653 at the C-terminus of human GRP 78. The HSP70-4 antibody is a rabbit monoclonal antibody raised against a synthetic peptide corresponding to residues of the human HSP70-4. β-actin antibodies and normal mouse IgG were purchased from Upstate Biotechnology, Inc. (Lake Placid, NY). Secondary antibodies used in these experiments were horseradish peroxidase-linked anti-mouse immunoglobulin G and anti-rabbit immunoglobulin G and purchased from Santa Cruz Biotechnology, Inc. Immunoblotting detection regents were purchased from Amersham Pharmacia Biotech (Piscataway, NJ). Dimethyl sulfoxide (DMSO), fluorescein isothiocyanate (FITC) antibody, 4′,6-diamidino-2-phenylindole (DAPI), and 3-(4,5-dimethylthiazol-2-yl)-5-(3-carboxyme-thoxyphenyl)- 2-(4-ulfophenyl)-2H-tetrazolium (MTT), were purchased from Sigma-Aldrich.

### Cell Culture and DNP Treatment

Human NPC cell lines 5-8F and 6-10B were purchased from the Cancer Research Institute of Sun Yatsen University (Guangzhou, China). The 5-8F cell line is highly metastatic, while the 6-10B cell line is only slightly metastatic [35]. The human NPC cell lines CNE1, CNE2, HNE1, HNE2, and HONE1 were purchased from the Cell Center of Central South University (Hunan, China). Cell lines were cultured in RPMI 1640 medium containing 10% fetal bovine serum (FBS), 2 mM l L-glutamine, 100 µg/ml penicillin, and 100 IU/ml streptomycin (Invitrogen, Carlsbad, CA), and were maintained in an incubator at 37°C and 5% CO_2_. For DNP treatment, DNP crystals were dissolved in DMSO, and the appropriate amounts of DNP stock solution were added to the cultured cells to achieve the indicated concentrations. The cells were then incubated for specified amount of time. To investigate the dose-course dependency of DNP treatment, cells were treated with 2 or 4 µM DNP for 24 h. For time-course assays, cells were treated with 4 µM DNP for 12 and 24 h [33].

### MTT Assay

To determine the non-cytotoxic concentration (NCC) of DNP to 6-10B cells, cells were treated and we performed an MTT assay to assess cell viability [34]. Briefly, 6-10B cells were seeded in 96-well plates at a density of 3.5×10^3^ cells/well, and then treated with 0 to ∼60 µM DNP for 24 h at 37°C. After the exposure period, media were removed and the cells were washed with phosphate-buffered saline (PBS). Thereafter, media were changed, and cells were incubated with 100 µl MTT (5 mg/ml) per well for 4 h. The viable number of cells per dish was directly proportional to the production of formazan, which was solubilized in isopropanol and measured using a spectrophotometer at 563 nm.

### LDH Assay

To further confirm the NCC of DNP in NPC cells, the LDH activity in cell culture media was measured after DNP treatment. Briefly, 6-10B cells were seeded in 6–well plates at a density of 2×10^4^ cells/well and treated with 0–10 µM DNP for 24 h at 37°C. After the exposure treatmentperiod, media were was collected for lactate dehydrogenase (LDH) activity measurement using the LDH assay kit (Autec Diagnostica).

### Immunofluorescence Analysis

Immunofluorescence analysis was performed as previously described [34]. Cells were fixed with formaldehyde in phosphate-buffered saline (PBS) for 30 min, and treated with PBS containing 0.2% Triton X-100 for 10 min after being washed with PBS. The cells were incubated with 0.5% bovine serum albumin in PBS, and incubated with HSP70-2 mouse antibody (Sigma-Aldrich) after being washed, and then incubated with the anti-mouse fluorescein isothiocyanate (FITC)-IgG antibody. Cells were again washed using PBS, mounted onto coverslips and examined under a Zeiss Axiophot microscope (Carl Zeiss, Oberkochen, Germany). Cells incubated with a non-specific IgG served as the blank control. 5-8F cells served as positive cells. Cells stained with DAPI served as the cell control.

### Western Blotting

Western blotting analysis was performed as previously described [33]. Briefly, cell samples were disrupted with lysis buffer. The sample protein concentration was determined using the BCA Protein Assay kit (Bio-Rad Laboratories, Inc., Hercules, CA). 40 µg protein was separated on a 10% or 12% polyacrylamide gel and transferred onto a nitrocellulose membrane. The blot was subsequently incubated with 5% non-fat milk to block non-specific binding, followed by incubation with the primary antibodies, and then with a peroxidase-conjugated secondary antibody. The signal was developed using 4-chloro-1-napthol/3,3-o-diaminobenzidine, and the relative photographic density was quantified using a gel documentation and analysis system. β-actin was used as an internal control. The abundance ratio to β-actin was then quantified.

### Reverse Transcription PCR (RT-PCR) and Quantitative Real-time PCR (qRT-PCR)

RNA analysis was performed as previously described [9]. RNA was harvested from the cells, and single-stranded cDNA synthesis was generated using the TaqMan RT Kit (Roche) with oligo-(dT)16 primers. The cDNA was used as the template for duplex PCR reactions that were performed by amplification of the housekeeping gene *GAPDH* together with the gene of interest. Primers used are as follows: P1, 5′-CCTACTCGGACAACCAGAG-3′; P2, 5′-TCTCGTCTTCCACCG TCTG-3′ [9]. PCR products were separated by 1.5% agarose gel electrophoresis according to standard protocol. Real-time PCR assay was performed using the ABI7900 system (Applied Biosystems) with TaqMan minor groove binder (MGB) probes (P3, 5′-TGTAGTACAACCGATATGTTCATTAGAATTC-3′; P4, 5′-TGGCAGTGTT GATTCGTTTAAAGG-3′; probe, 5′-TAATGTTGATACTGTAAGGGTG-3′) [36]. The housekeeping gene, GAPDH served as a loading control, H_2_O was used as the blank control.

### Construction of Expression Vectors

A 1,176 bp *HSP70-2* promoter construct (21,114/162) was generated from human genomic DNA using PCR. The primers used were P3 (5′-TTGTGAGCTCTGGAGTGTAGTGGCG TGAT-3′) and P4 (5′-TTGTAAGCTTGACG CACGAATAGGTGGT-3′). The PCR product was cloned into *Sac*I and *Hind*III sites of pGL3-Basic vector, to generate the pGL3-*HSP70-2* construct. The resulting construct was confirmed by DNA sequencing. pGL3*-HSP70-2*M1 (HRE1 mutation) was generated using the pGL3*-HSP70-2* construct as a template, and P3 and P5 (5′-GCCAATCCGATTAGAGTCGGCTAGG-3′) primers. pGL3*-HSP70-2*M2 (HRE1, HRE2 mutation) was generated using pGL3*-Hsp70-2*M1 as a template, and P6 (5′-TCTGTTGGGTTCTAGGCCAGTCACT-3′) and P7 (5′-AGTGACTGGCCTAGA ACCCAACAGA-3′) primers. pGL3*-Hsp70-2*M3 (HRE1, HRE2, HSE mutation) was generated using pGL3*-Hsp70-2*M2 as a template and primers P8 (5′-TGGGATTAAATCT GGGAGTT-3′) and P9 (5′-AACTCCCAGATTTAATCCCA-3′); P10 (5′-AGCCCGAATGCTTGGTTCG-3′) and P11 (5′-CGAACCAAGCATTCGGGCT-3′) [37]. Additionally, the *HSP70-2* DNA fragment was generated by PCR and cloned into the *Bam*HI/*Xho*I site of the pcDNA3.1 vector (Amersham Biosciences) to generate pcDNA3.1-*HSP70-2* plasmids. siRNA was designed against the mRNA sequences targeting *HSP70-2* (A2.2): 5′-GGACAUUGGGCCCAACAAG-3′. The pU6pro vector was used to generate the pU6pro-si-mock (si-mock) and pU6prosi-HSP70-2 (si-*HSP70-2*) plasmids following the manufacturer′s protocol [9].

### In *vitro* Binding Assay of DNP and the HSP70-2 Promoter

pGL3-*HSP70-2*, pGL3-*HSP70-2*M1, pGL3-*HSP70-2*M2, and pGL3-*HSP70-2*M3 constructs were each transformed into BL21 *Escherichia coli* cells. The transformed BL21 *Escherichia coli* were then grown and the DNA samples were extracted from the transfected bacteria and purified. Plasmid DNA (5 µg) was incubated with 1 µCi ^3^H-DNP at 4 °C overnight. These reactive products were boiled and separated by SDS-PAGE for autoradiography. Further, the affinity of DNP for the HSP70-2 promoter was determined by displacement of [^3^H]-DNP from the HSP70-2 promoter DNA *in vitro* as previously described [38] with modifications. HSP70-2 promoter DNA (30 µg) plus ^3^H-DNP and unlabeled DNP at various concentration were incubated for 1 h at 30°C. Samples were filtered over glass fiber GF/B filters using a Tomtec harvester and radioactivity was quantified using a Wallac Betaplate counter as previously described [38]. The apparent affinity of DNP for the HSP70-2 promoter was then determined.

### Reporter Gene Assays

The reporter gene assay for to quantify firefly luciferase activity was performed using lysates from transfected cells [39]. Briefly, the reporter gene vectors, pGL3-*HSP70-2*, pGL3-*HSP70-2*M1, pGL3-*HSP70-2*M2, or pGL3-*HSP70-2*M3 was transfected into 6-10B cells and *Renilla* luciferase activity was used to normalize for transfection efficiency. Lysis buffer (100 µl) was then added to the transfected cells (Promega Dual Luciferase Reporter Assay System). Firefly and *Renilla* luciferase activity was measured in the lysate supernatant. Next, 20 µl cell lysate was mixed with 100 µl Luciferase Assay II reagent and firefly luciferase emission was measured by using a Luminoskan Ascent plate reader (Thermo Electron Corp., Helsinki, Finland). Subsequently, coelenterazine reagent (100 µl) containing the *Renilla* luciferase substrate was mixed to normalize the firefly luciferase data. The results were expressed as relative transcript activity of *HSP70-2* promoter (fold) as described previously [39].

### Cell Motility and Invasion Assays

For the cell invasion assay, 6-10B cells were treated with the indicated concentrations of DNP for the indicated times. After DNP treatment, cells were trypsinization and 1.5×10^4^ cells/well in 50 µl of serum-free medium were seeded into the upper part of a Boyden chamber (Neuro Probe, Cabin John, MD) coated with Matrigel for *in vitro* measurement of invasiveness [40]. The bottom chamber also contained standard medium with 20% FBS. Cells were analyzed for invasiveness after 12 h incubation at 37°C. Specifically, cells that invaded the lower surface of the membrane were fixed with methanol and stained with hematoxylin and eosin. The number of cells in random fields of view were counted under a light microscope and deemed “invading cells.” To determine cell motility, the treated cells were seeded into the Boyden Chamber on membrane filters that were not coated with Matrigel. Migration of cells was measured as described in the motility assay [40]. Statistical analysis was corrected with cell viability to clarify normalize the effects of DNP treatment.

### Animals

A total of 20 female nude BABL/c mice (approximately 5–6-week-old) were purchased from the Animal Center of Central South University. They were maintained in the Laboratory for Experiments, Central South University under laminar airflow conditions. The studies were conducted according to the standards established by the Guidelines for the Care and Use of Laboratory by Animals of Central South University.

### Effect of DNP on NPC Metastasis in Nude Mice

The metastatic effect of DNP on 6-10B cells was determined *in vivo* as previously described [41]. Briefly, 100 µl aliquots of 6-10B cell suspensions (1×10^4^ cells) were mixed with Matrigel. The cell suspensions were injected into the tail veins of the 20 nude mice, and then randomly divided into 2 groups, containing 10 mice per group. The DNP-treated group was abdominally injected with DNP at a dose of 40 mg/kg (body weight) twice a week for 60 days, and the control was injected 0.1% DMSO [33]. Metastasis of 6-10B tumor cells to lymph nodes was determined after DNP treatment. Metastasis was evaluated by measuring the weight of metastasized tumors.

### Immunohistochemistry

HSP70-2 protein levels were evaluated by examining 4 µm thick, paraffin-embedded tissue sections and HSP70-2 antibodies or IgG controls as described previously [42]. HSP70-2 immunostaining of a stained tumor region was examined under a 400× magnification as previously described [43]. Immunoreactivity was considered positive in specimens where >10% of cancer cells were HSP70-2immunoreactive.

### Gene Transfection and Stably Transfected Cell Lines

6-10B cells were transfected with pcDNA3.1 (mock), pU6pro-si-*HSP70-2,* or pcDNA3.1-*HSP70-2* using Lipofectamine 2000 reagent (Life Technologies, Inc.) following the manufacturer’s suggested protocol. The stably transfected cell lines were obtained by selection for G418 resistance (400 μg/ml) as described previously [34] and further confirmed by assessing HSP70-2 expression. To confirm whether DNP promotes metastasis through HSP70-2, 6-10B-si-*HSP70-2* cells were treated with DNP, and their motility and invasiveness was quantified using the *in vitro* Boyden chamber invasion assay.

## Results

### The Non-cytotoxic Concentration (NCC) of DNP in 6-10B NPC Cells

**Figure 1:**
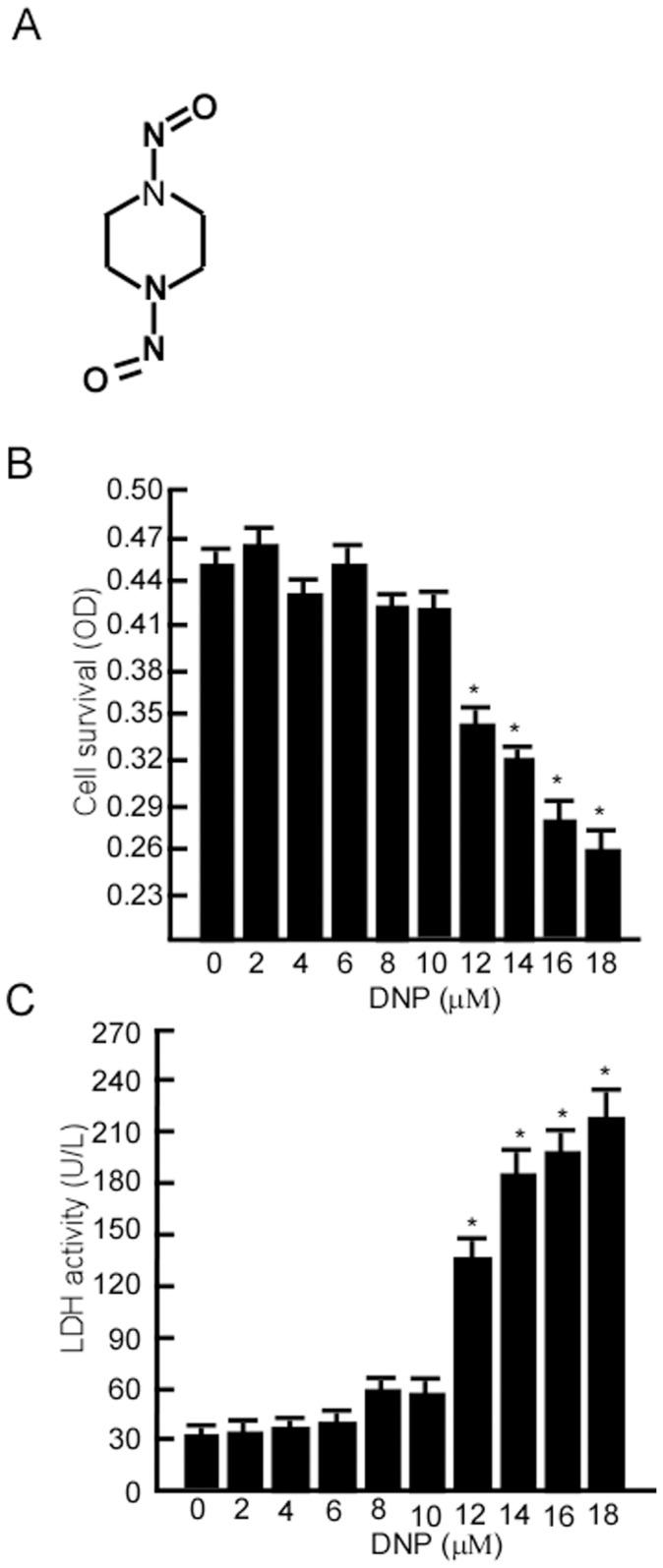
Non-cytotoxic concentration of *N*,*N*′-dinitrosopiperazine (DNP) in 6-10B cells. A, Structure of DNP, an *N*-nitroso compound. B, 6-10B cells were treated with the indicated concentration DNP for 48 h before being subjected to the MTT cell viability assay. C, After 6-10B cells were treated with the indicated DNP concentration for 48 h, cell media were subjected to the LDH assay. The optical density (OD) indicates the relative OD at 563 nm. Lactate dehydrogenase (LDH) activity is expressed per 1 L of media. Data are presented as means ± SD from 3 independent experiments. Statistical analysis was performed using the Student’s *t* test (*, *p*<0.05).

DNP is an important carcinogenic *N*-nitroso compound for NPC and its chemical structure is shown in Fig. 1A. In this study, we first determined the NCC of DNP by treating 6-10B cells with various DNP concentrations for 48 h, and then subjecting the cells to the MTT assay. Compared with the control (0.1% DMSO), in DNP treatment, the cell viability was not significantly altered at 0.5–6 µM (Fig. 1B; *, *p*<0.05). To further validate that 0.5–10 µM DNP was non-cytotoxic, lactate dehydrogenase (LDH) activity in the cell culture media was measured after DNP treatment. The data revealed that LDH activity was not significantly altered by treatment with DNP concentrations of 0.5 and 6 µM (Fig. 1C; *, *p*<0.05). Thus, this NCC range was used in all subsequent experiments.

### DNP Induces HSP70-2 Expression in a Dose-dependent and Time-dependent Manner

**Figure 2:**
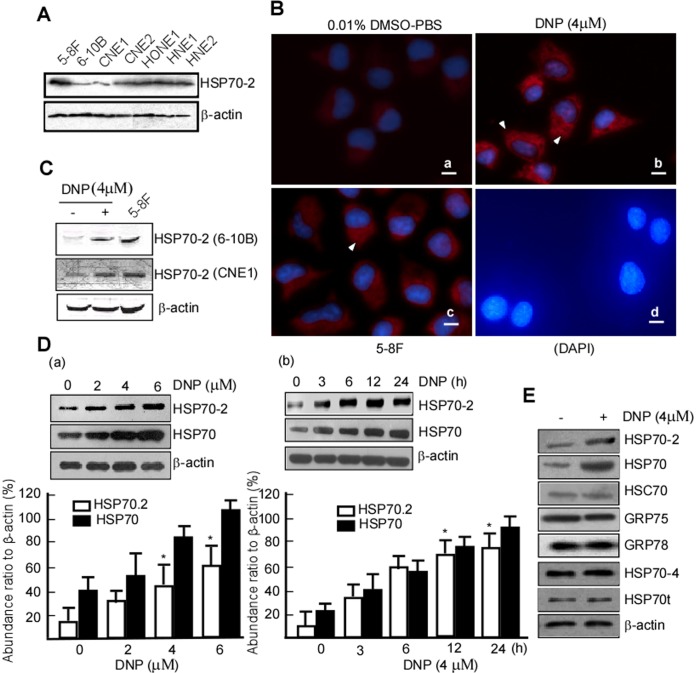
Dose- and time- dependent DNP-mediated HSP70 expression. A, HSP70-2 expression was detected in NPC cell lines 5-8F, 6-10B, CNE1, CNE2, HONE1, HNE1, and HNE2 by Western blotting. B, DNP-mediated HSP70-2 expression was detected using immunofluorescence. a, 6-10B cells treated with 0.1% DMSO-PBS served as a negative control; b, 6-10B cells treated with DNP; c, 5-8F served as a positive control; d, 6-10B cells stained with DAPI. Scale bar = 50 µm. C, DNP-mediated HSP70-2 expression was detected in 6-10B and CNE2 cells. D, Time- and dose-dependency of DNP-induced HSP70-2 expression. a, HSP70-2 expression in 6-10B cells treated with the indicated concentration; b, HSP70-2 expression in 6-10B cells with 4 µM DNP for the indicated time. Three independent experiments were performed, the abundance ratio to β-actin was counted, and data are represented as means ± SD from three experiments. *, p<0.05. E, Expression of HSP70-2, HSP70t, HSC70, GRP75, GRP78, and HSP70-4 was detected in 6-10B cells after DNP treatment.

To investigate the mechanism of DNP-mediated HSP70-2 expression, NPC cell lines with low levels of HSP70-2 were screened through Western blot analysis of NPC cell lines 6-10B, 5-8F, CNE1, CNE2, HONE1, HNE1, and HNE2. Our results show that 6-10B and CNE1 cells express low levels of HSP70-2 (Fig. 2A). As such, 6-10B and CNE1 cell lines were used to investigate DNP-mediated HSP70-2 expression. Immunostaining revealed that HSP70-2 is predominantly localized to the cytoplasm and was enhanced in 6-10B cells with after DNP treatment as compared with to controls (Fig. 2B, b *vs.* a). Western blotting confirmed that HSP70-2 expression was increased after DNP treatment (Fig. 2C, lane 2 *vs.* 1, upper panel). This result was confirmed in CNE2 cells (Fig. 2C, lane 2 *vs.* 1, middle panel). These findings indicate that DNP can induce HSP70-2 expression. To further investigate the dose and time course of DNP-induced HSP70-2 expression, the cells were treated with approximately 2–6 μM DNP or with 4 μM DNP for approximately 12–24 h, and then HSP70-2 expression was examined. DNP-mediated HSP70-2 induction is both dose and time-dependent (Fig. 2D a-b). The HSP70 family consists of at least 8 highly homologous members, so we next analyzed whether DNP induces expression of the other HSP70 family members HSP70t, HSC70, GRP75, GRP78, and HSP70-4, DNP did not induce expression of these targets (Fig. 2E, lane 1 *vs.* 2 in panel 3, 4, 5, 6, 7, and 8); only HSP70-2 expression was increased following DNP treatment (Fig. 2E, lane 1 *vs.* 2, panel 1). Thus, DNP only induces HSP70-2 expression and not other protein family members.

### DNP Induces *HSP70-2* Transcription through Binding to its Promoter, HER1, 2 and HSE

**Figure 3:**
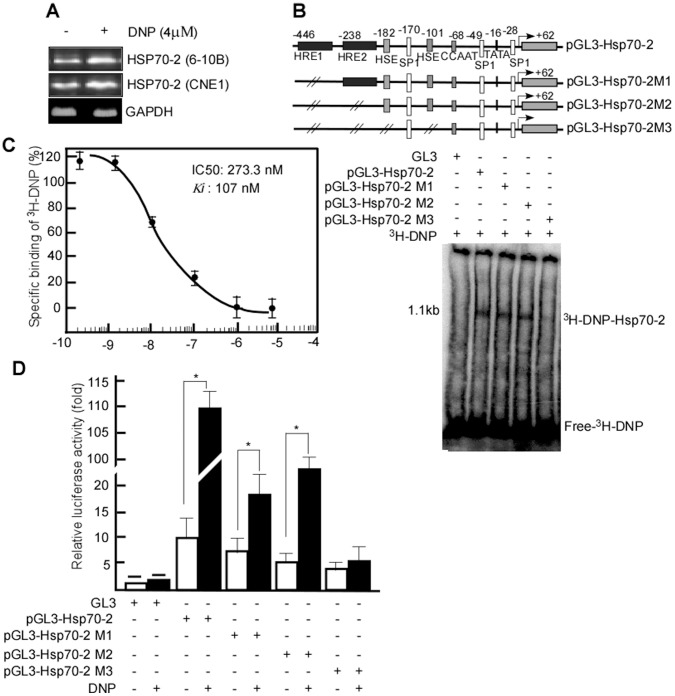
DNP-mediated HSP70 RNA transcription through HSP70-2 promoter interaction. A, DNP-mediated Hsp70 transcription was detected in 6-10B and CNE1 cells by RT-PCR. B, Schematic of the HSP70-2 promoter and its mutations. HSP70-2 promoter, the full length of promoter; HSP70-2M1, HER1 promoter mutation; HSP70-2M2, HER1, and HER2 mutation; HSP70-2M3, HER1, 2 and HSE mutation. HSP70-2 promoter and its mutations were respectively incubated with ^3^H-DNP, and the reactive complexes were separated by SDS-PAGE for autoradiography. C, The affinity was determined by displacement of ^3^H-DNP from the HSP70-2 promoter. IC50 of DNP interaction with HSP70-2 promoter was 273.3 nM, and Ki was 107 nM. D, Relative firefly luciferase activities were normalized by the Renilla luciferase activity (mean ± SD). The relative luciferase activity indicates the transcriptional activity of the HSP70-2 promoter and its mutation with or without DNP treatment.

To further determine whether the induction of HSP70-2 expression by DNP occurs through the upregulation of HSP70-2 RNA, the HSP70-2 RNA was quantified in 6-10B and CNE2 cells treated with DNP by using RT-PCR. Following DNP treatment, HSP70-2 RNA expression increased (Fig. 3A, lane 1 *vs.* 2 upper panel), and similar results were obtained in CNE2 cells (Fig. 3A, lane 1 *vs.* 2, middle panel). DNP-induced HSP70-2 RNA transcription was further validated using real-time PCR. HSP70-2 RNA copy number was (6.81±2.37)×10^7^ in the cells with DNP treatment, which was higher than untreated cells (2.77±1.07)×10^4^ (*p*<0.05). These data indicated that DNP induces HSP70-2 RNA transcription.

HREs and HSE are critical elements to HSP70-2 RNA transcription. Therefore, we next investigated whether DNP binds to these elements to regulate HSP70-2 transcription. Reporter gene constructs of the *HSP70-2* promoter and its mutations, pGL3*-HSP70-2,* pGL3*-HSP70-2*M1, pGL3*-Hsp70-2*M2 and pGL3*-HSP70-2*M3 were constructed. The interactions of *HSP70-2* promoter and its mutations with ^3^H-DNP were examined. These results indicate that DNP could bind to the *HSP70-2* promoter, *HSP70-2*M1 *and HSP70-2*M2, but not to *HSP70-2*M3 (Fig. 3C). This suggests that DNP interacts with the *HSP70-2* promoter *via* HER1, 2 and/or HSE. To further investigate DNP binding to the HSP70-2 promoter, a competitive binding assay was performed. The results indicate that the IC50 of DNP interaction with HSP70-2 promoter was 273.3 nM, and the Ki was 107 nM (Fig. 3D). This suggests that DNP covalently binds to the HSP70-2 promoter.

To determine whether DNP-binding to the *HSP70-2* promoter affects RNA transcription, transcript activities of pGL3*-HSP70-2,* pGL3*-HSP70-2*M1, pGL3*-HSP70-2*M2, *and* pGL3*-HSP70-2*M3 were measured by firefly luciferase expression after DNP treatment. pGL3*-HSP70-2* construct had 11 folds high luciferase expression after DNP treatment, pGL3-*HSP70-2*M1 exhibited a 2.2-folds higher expression, pGL3-*HSP70-2*M2 exhibited a 3.8-fold higher expression, but pGL3-*HSP70-2*M3 showed no significant expression changes (Fig. 3E). These data suggest that DNP may induce transcription of *HSP70-2* through binding to HER1, 2 and HSE.

### DNP Induces Invasion and Motility of 6-10B Cells

**Figure 4:**
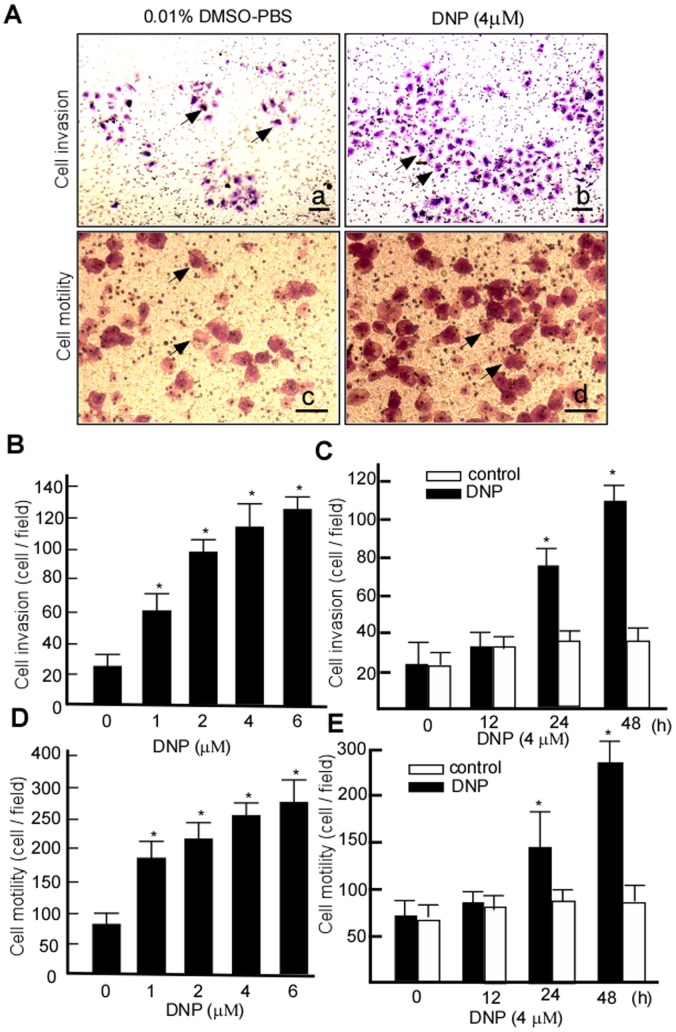
DNP-mediated NPC cell metastasis *in vitro*. A, Matrigel-coated Boyden chambers was used to measure 6-10B cell invasion, and an uncoated Boyden chamber was used to determine cell motility after DNP treatment. The cells invaded to the lower surface of the membrane were fixed with methanol and stained with hematoxylin and eosin. a and c, 6-10B cells treated with 0.1% DMSO-PBS; b and d, 6-10B cells treated with 4 µM DNP; Scale bar = 10 µm. Arrows = invaded cell. In dose-dependency assays, 6-10B cells were treated with the indicated concentrations DNP. In time-course assays, cells were treated with 4 µM DNP for the indicated time. Treated cells were subjected to motility and invasion analysis. Images were taken under a light microscope and random fields of view were counted to determine the number of invading cells. B, Invasion of 6-10B cells at various concentrations; C, Invasion of 6-10B cells at various time points; D, Motility of 6-10B cells at various concentrations; and E, Motility of 6-10B cells at various time points. Statistically analysis performed using a one-way analysis of variance (ANOVA) with Dunnett’s post-hoc test (*, p<0.05).

We next determined if DNP could induce cell invasion and motility of using Boyden chambers. After DNP treatment, invading cells dramatically increased in the Matrigel-coated Boyden chamber (Fig. 4A-b) and the uncoated Boyden chamber (Fig. 4A–d). The invading cells increased in a dose-dependent manner (Fig. 4B, *, *p*<0.05), and the increase was 421.7% with 6 µM DNP (Fig. 4B, lane 5). A similar effect was observed for the motility of DNP-treated cells (Fig. 4D, *, *p*<0.05), and the increase was 328.2% after treatment with 6 µM DNP (Fig. 4D, lane 5). DNP increased invasion (Fig. 4C, *, *p*<0.05) and motility (Fig. 4E, *, *p*<0.05) in a time-dependent manner with the effects prolonged to 48 h. These findings indicate that DNP can induce NPC cell motility and invasion.

### DNP-mediated Invasion and Motility Occurred through Increased-HSP70-2 Expression

**Figure 5:**
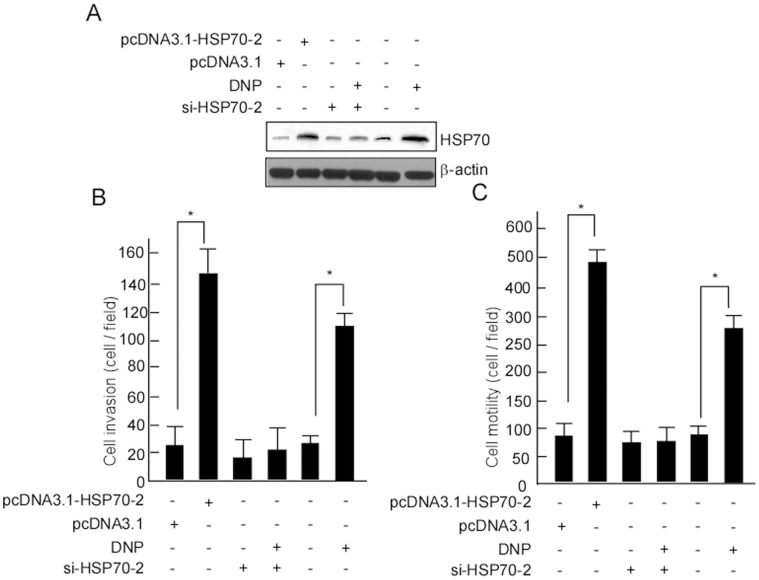
DNP induces motility and invasion through HSP70-2. A, 6-10B cells were transfected using pcDNA3.1-HSP70-2, pcDNA3.1, pU6pro-si-HSP70-2, or pU6pro-si-mock, and then the stable cell lines 6-10B-HSP70-2, 6-10B -si-HSP70-2, 6-10B-pcDNA3.1, and 6-10B-si-mock were obtained by selection for G418 resistance. HSP70-2HSP70-2 expression was detected in these cells with or without DNP treatment. β-actin is shown as a loading control. The motility (B) and invasion (C) of 6-10B - HSP70-2, 6-10B-si-HSP70-2, 6-10B-pcDNA3.1, and 6-10B-si-mock with or without DNP treatment were detected. Data are presented as means ± SD from 3 independent experiments. Results were analyzed by One-way ANOVA with post-hoc Dunnett’s test (*, p<0.05).

To confirm whether NPC metastasis occurs through HSP70-2, 6-10B cells with low levels of HSP70-2 and metastatic potential were transfected with pcDNA3.1-HSP70-2, its motility and invasion were dramatically increased after being transfected (Fig. 5B, *, *p*<0.05). Cells were then transfected using pU6pro-si-HSP70-2, and a stably transfected cell line 6-10B-si-HSP70-2 was obtained. HSP70-2 was silenced in the 6-10B-si-HSP70 cells (Fig. 5A, lane 3 in upper panel). To further confirm whether DNP induces cell motility and invasion through HSP70-2, motility and invasion were examined in 6-10B-si-HSP70 cells with and without DNP treatment. DNP-induced motility and invasion was not altered when HSP70-2 expression was blocked (Fig. 5B, lane 3, 4; Fig. 5C, lane 3, 4); however, DNP could effectively induce cell motility (Fig. 5C, lane 1 *vs.* 2, *p*<0.05; Fig. 5C, lane 5 *vs.* 6, *p*<0.05) and invasion (Fig. 5B, lane 1 *vs.* 2, *p*<0.05; Fig. 5B, lane 5 *vs.* 6, *p*<0.05) when HSP70-2 was not blocked. Together, our data indicate that DNP induces cell metastasis via induction of HSP70-2.

### DNP-mediated Metastasis *via* HSP70-2 Confirmed *in vivo*

**Figure 6:**
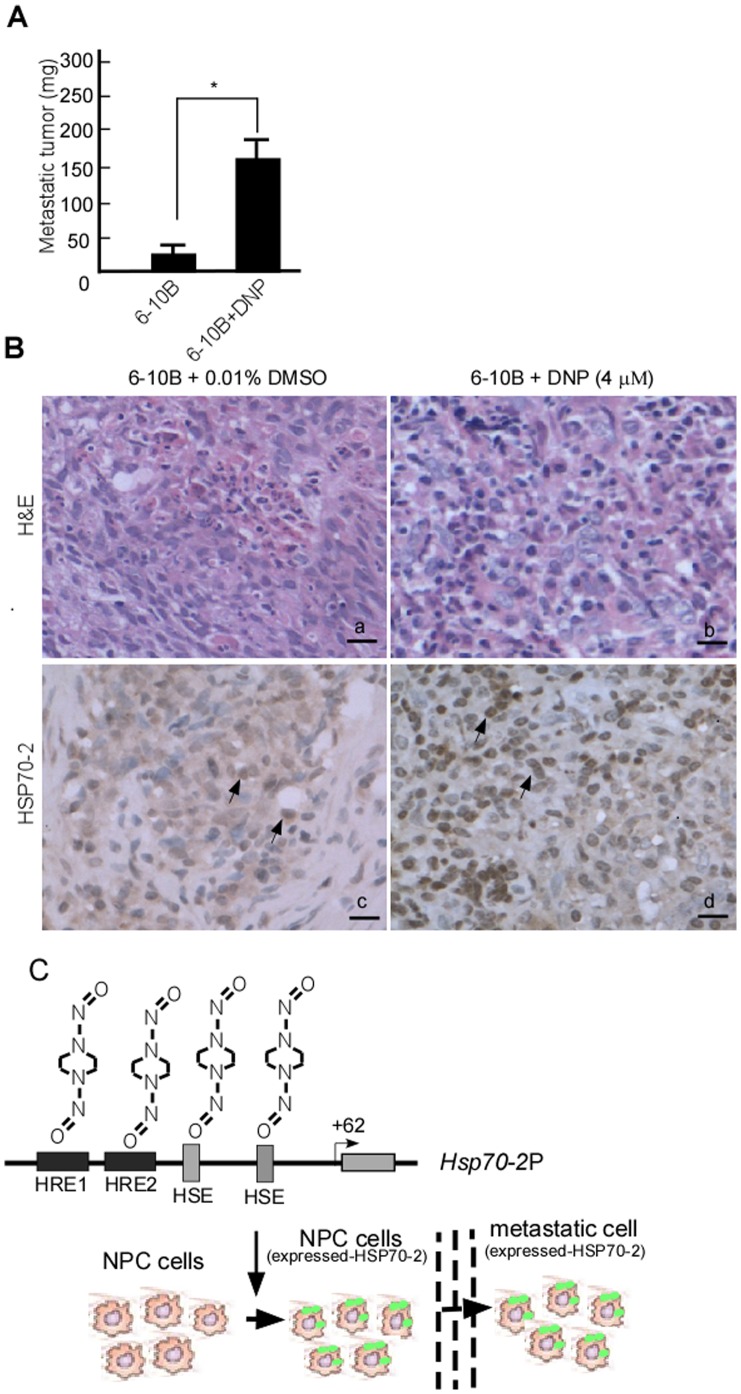
DNP-mediated NPC cell metastasis and HSP70-2 expression *in vivo*. A, 20 BABL/c nude mice were injected with 6-10B cells in Matrigel through the tail vein (1×10^4^ cells/mouse), and randomly divided into 2 groups (DNP-treated and control groups) with 10 mice per group. The DNP-treated group was abdominally injected using DNP at a dose of 40 mg/kg twice a week for 60 days. The control group was injected using 0.1% DMSO-PBS. After 60 days, metastatic tumors from the mediastinal lymph nodes were weighed (*, p<0.05). B, HSP70-2 expression was detected in the metastatic tumor samples using immunohistochemistry. Paraffin sections were stained using hematoxylin and eosin as well as with antibodies against HSP70-2. The upper panel represents staining with hematoxylin and eosin; the lower panel represents immunohistochemistry data. a and c represent untreated 6-10B cells as a negative control; b and d represent DNP-treated 6-10B cells; Arrows = positive cell. Original magnification, ×400. Scale bar = 5 µm. C, Schematic illustration of DNP-induced HSP70-2. DNP-mediated HSP70-2 expression through binding to HSP70-2 promoter, promotes motility and invasion, leading to metastasis of NPC cells.

The metastasis-inducing effects of DNP were confirmed in nude mice. 6-10B cells were mixed with Matrigel, and then injected into the tail veins of BABL/c nude mice. The nude mice were treated using DNP for 60 days. To compare with the control, metastasis of 6-10B cells to the mediastinal lymph nodes significantly increased in the DNP treated group (Fig. 6A, lane 2 *vs.* 1; *p*<0.05). To determine whether the metastasis-inducing effects of DNP were associated with HSP70-2, the HSP70-2 levels were analyzed in the metastatic tumors by using immunohistochemistry. HSP70-2 levels were higher in metastatic tumors from DNP-treated mice than in those from untreated control mice (Fig. 6B, a *vs.* b).

## Discussion

NPC exhibits highly invasive and metastatic features, and approximately 90% of patients show cervical lymph node metastasis at the time of initial diagnosis [2]. As one well-established carcinogenic factors, EBV is involved in NPC metastasis, and EBV latent membrane protein (LMP) 1 and 2 have been shown to promote progression and metastasis of NPC; LMP was positive at 56% [44], [45]. This could be due to the fact that it has not yet fully adopted the high invasiveness and metastatic nature of NPC. Recently, another NPC carcinogen, DNP has shown to be involved in NPC metastasis. Studies that have examined DNP-induced NPC in rats, have found that DNP exhibits organ specificity to the nasopharyngeal epithelium and a high metastasis incidence [6], [31]. In the clinic, NPC patients with metastasis exhibit high DNP levels as compared with to those without metastasis. From previous work and the data presented here [33], we believe that DNP is involved in NPC metastasis by examination of three lines of evidence. First, NPC patients with metastasis have higher serum DNP levels that those without metastasis. Second, DNP can induce NPC cell motility and invasion *in vitro*. Third, DNP can increase NPC cell metastasis of mediastinal lymph nodes *in vivo*. Additionally, In nasopharyngeal carcinogenesis, DNP and EBV have a synergistic effect [46]. Based on this, we speculate that DNP and EBV also have synergy effect in NPC metastasis.

HSP70 expression in NPC is correlated with IgA titers against the viral capsid antigen of EBV in the sera of NPC patients. HSP70 expression in stage II–III of NPC tissues is positively correlated to EBV IgA/VCA titers. The prognosis of HSP70-positive NPC patients is poor [47]. The EBV viral antigen, EBNA3A, induces several chaperone molecules, including HSP70, HSP70B/B′, and HSP40 [48]. DNP may also regulate HSP70-2 through EBV viral antigen expression. Our previous work has shown that DNP induces rat NPC with high metastasis and is accompanied by high HSP70 expression [6]. The human HSP70 family consists of at least 8 highly homologous members that differ from each other based off intracellular localization and expression patterns [49]. In the present study, DNP only induce HSP70-2 expression and did not effect other family members such as, HSP70t, HSC70, GRP75, GRP78, and HSP70-4. We show that DNP induced *HSP70-2* RNA transcription acts through DNP binding to the HER1, 2 and HSE promoter. HSP70-2 is upregulated in metastatic cancers and its expression promotes cancer metastasis. HSP70-2 knockdown significantly suppressed cellular motility and invasion ability [10], [50]. Our data shows that DNP induced HSP70-2 expression and promoted motility and invasion of NPC cells. Moreover, high HSP70-2 expression in metastatic tumors is induced by DNP. We speculated that DNP might induce NPC metastasis through the regulation of HSP70-2. To further validate this hypothesis, we established stably transfected cell lines containing HSP70-2 (that overexpress HSP70-2) or si-HSP70-2 (block HSP70 expression), and observed the effects of DNP on cell motility and invasion. Interestingly, cell motility and invasion was dramatically increased when transfected using HSP70-2 or DNP treatment while DNP-mediated motility and invasion ability did not alter when HSP70-2 expression was silenced.

As a molecular chaperone, HSP70-2 plays an important role in protein-protein interactions that result in the proper folding, confirmation, transport, and assembly of proteins in the cytoplasm, mitochondria, and endoplasmic reticulum [7], [8]. HSP70-2 may be involved in various cellular functions such as cell adhesion and MMP-2 activation. DNP-mediated HSP70-2 induction may also be involved in cell metastasis through increased cell adhesion, migration, and activation of MMP-2. However, these statements need to be tested in future studies. Here, we provide 3 lines of evidence that support DNPs involvement in NPC metastasis through the regulation of HSP70-2. First, DNP induces motility and invasion of 6-10B cells following HSP70-2 expression. Second, DNP enhances lymph node metastasis of NPC cells in nude mice and HSP70-2 is highly expressed in metastatic lymph samples. Finally, silencing of HSP70-2 dramatically reduced DNP-mediated motility and invasion.

### Conclusion

DNP induces *HSP70-2* RNA transcription through binding to its promoter, HER1,2, and HSE. This interaction mediates HSP70-2 expression, and enhances cell motility and invasion, which are required for NPC cell metastasis (Fig. 6C).
